# Knowledge and risk perception of oral cavity and oropharyngeal cancer among non-medical university students

**DOI:** 10.1186/s40463-016-0120-z

**Published:** 2016-01-28

**Authors:** Nosayaba Osazuwa-Peters, Nhial T. Tutlam

**Affiliations:** Brown School, Washington University in St. Louis, 1 Brookings Drive, Saint Louis, MO 63130 USA; Saint Louis University Cancer Center, 3655 Vista Avenue, Saint Louis, Missouri 63110 USA; Department of Otolaryngology-Head and Neck Surgery, Saint Louis University, School of Medicine, 6th Floor Desloge Building, 3635 Vista Avenue, Saint Louis, MO 63110 USA; Department of Epidemiology, Saint Louis University, College for Public Health and Social Justice, 3545 Lafayette Avenue, Saint Louis, Missouri 63108 USA

**Keywords:** Oral cavity and oropharyngeal cancer, non-medical university students, knowledge, risk perception, sexual habits

## Abstract

**Background:**

To assess non-medical university students' knowledge and perceived risk of developing oral cavity and oropharyngeal cancer.

**Methods:**

A cross-sectional survey was conducted among non-medical students of a private Midwestern university in the United States in May 2012. Questionnaire assessed demographic information and contained 21 previously validated questions regarding knowledge and perceived risk of developing oral cavity and oropharyngeal cancer. Knowledge scale was categorized into low and high. Risk level was estimated based on smoking, drinking, and sexual habits. Bivariate associations between continuous and categorical variables were assessed using Pearson correlation and Chi-square tests, respectively.

**Results:**

The response rate was 87% (100 out of 115 students approached). Eighty-one percent (81%) had low oral cavity and oropharyngeal cancer knowledge; and only 2% perceived that their oral cavity and oropharyngeal cancer risk was high. Risk perception was negatively correlated with age at sexual debut, *r* (64) = −0.26, *p* = 0.037; one-way ANOVA showed a marginally significant association between risk perception and number of sexual partners, *F*(4, 60) = 2.48, *p* = 0.05. There was no significant association between knowledge and perception of risk; however, oral cavity and oropharyngeal cancer knowledge was significantly associated with frequency of prevention of STDs (*p* < 0.05). Although 86% had heard about oral cavity and oropharyngeal cancer, only 18% had heard of oral mouth examination, and 7% of these reported ever having an oral cavity and oropharyngeal cancer exam.

**Conclusions:**

Oral cavity and oropharyngeal cancer knowledge and risk perception is low among this student population. Since oral cavity and oropharyngeal cancer incidence is increasingly shifting towards younger adults, interventions must be tailored to this group in order to improve prevention and control.

## Background

Oral cavity and oropharyngeal cancer is found around the oral cavity and oropharynx, and is the 6th most common cancer in the world, with estimated annual incidence of more than 405, 000 cases [1]. In 2012 in the United States, it was estimated that about 40,250 new cases of oral cancers will be diagnosed [2], thus meeting qualification criteria to be regarded as a common cancer in the United States [3]. They account for almost 3% of all cancers, and are the 13th most common cancer in the United States [4, 5]. The causative factors linked to oral cancer include chronic use of tobacco and alcohol, which act independently and synergistically in the etiopathogenesis of the disease [1, 6]. An increasing number of cases of non-smoking, non-drinking individuals have helped to establish another major causal factor - human papillomavirus (HPV), which was thought to account for up to 23% of cases of oropharyngeal cancer [7]. A newer study however indicates that at least 70% of the oropharyngeal cancer incidence in the United States, in the last decade, may be causally linked to HPV [8].

Young adults of university age are known to engage in tobacco smoking and alcohol use, and may be frequently exposed to HPV infection, all of which are causal factors for oral cavity and oropharyngeal cancer [1, 6, 9]. Previous studies indicate that university students may have some knowledge of risks associated with tobacco smoking and alcohol [10–12]. However, while HPV remains the most prevalent sexually transmitted infection in the United States, knowledge of HPV among students remains quite low [13]. It has been reported that some college students even misinterpret HPV as HIV [13], and the latter remains a dominant topic of discussion in many sex education curricula across the United States [13, 14]. Over a decade ago in a Kaiser family survey, only 2% of the surveyed American population of young people not older than 18 years old, could identify that HPV was a sexually transmitted disease [14]. Knowledge of the link between oral cavity and oropharyngeal cancer and HPV seems even lower. Globally, the few studies that have examined students’ knowledge of oral cavity and oropharyngeal cancer only looked at medical and dental students [15–20], who may presumably present with better knowledge scores due to their health-directed training. The only known study that compared undergraduates to medical students and to the general population focused exclusively on HPV as a causal factor for head and neck cancer [21]. No other study, to the best of the author’s knowledge, has examined knowledge levels of oral cavity and oropharyngeal cancer exclusively among non-medical students, who may receive greater overall benefit from a customized health education program.

With the recent downward shift in the age of onset of oral cavity and oropharyngeal cancer, especially oropharyngeal cancer, from the typical sixth to seventh decade of life forty years ago [4, 8] to the third or fourth decade of life in the last twenty years [9, 22], there is a need for increased population based education about oral cavity and oropharyngeal cancer among university students, who represents a sizable population in the United States [23]. This study aims to examine non-medical university students’ level of knowledge of oral cavity and oropharyngeal cancer and its risk factors, and to determine university students’ perception of their risk of developing oral cavity and oropharyngeal cancer.

## Methods

### Study design

A cross-sectional study was conducted between May and June 2012 in a private, research university in the Midwest. Approval for this study was sought and obtained as protocol ID: 201205057 from the Institutional Review Board of the university prior to commencement of study.

### Study participants

Participants for the study included conveniently sampled male and female students in the university, both undergraduate and graduate students. Students were recruited to voluntarily participate in the study from the university main library and cafeteria. There was no compensation for participating in the study.

### Eligibility criteria

University students 18 years or older were eligible to take part in the study. In addition, participants needed to be able to consent to study in order to participate.

### Exclusion criteria

In order to assess the level of oral cavity cancer knowledge among non-medical university students, all medical, nursing, and public health students were excluded from the study. Additionally, all students who were previously enrolled in a head and neck cancer study, or for those students less than 18 years old, were excluded from the study. In addition, those who had been previously diagnosed with oral head and neck cancer were ineligible to participate.

### Data collection

#### Questionnaire

The study employed a previously validated 58-item, paper-based questionnaire adapted from previous studies [12, 15, 16, 19].

#### Demographic variables

The survey included demographic questions such as age, sex, relationship status, race, and educational level.

#### Risk factor-related variables

Students were asked *“Do you smoke?” “If yes, how many cigarettes do you smoke daily?”* and *“If you are a current smoker, how long have you been smoking?”* Additionally, students who were smokers were asked to gauge their relative risk of developing oral cavity and oropharyngeal cancer compared to other smokers of same age and sex. Students were also asked *“Do you drink alcohol?” “How often do you drink?” “If you still drink, how long have you been drinking?”* and *“If you have stopped drinking, when did you stop?”* Finally, students who drank alcohol were asked to gauge perceived oral cavity and oropharyngeal cancer risk.

To elicit risk factors for oral HPV, students were asked the following: “*Have you ever had sexual intercourse?”, “At what age did you first have sexual intercourse”, and “How many sexual partners have you had in your lifetime”.* Finally, students were asked how frequently they used STD prevention during sexual intercourse, on a scale from “never” to “always”.

#### Knowledge of oral cavity and oropharyngeal cancer

Students were asked 14 questions to gauge their knowledge of oral cavity and oropharyngeal cancer. General questions that elicited awareness included *“In your opinion, oral cavity and oropharyngeal cancer is more common in which age group?” “In your opinion, in which gender is oral cavity and oropharyngeal cancer more common?* and *“Does early diagnosis improve recovery from oral cavity and oropharyngeal cancer?”* Additionally, some questions focused on the pathophysiology of oral cavity and oropharyngeal cancer, such as: *“In your opinion, where are the most likely locations of oral cavity and oropharyngeal cancer?” “In your opinion, which of these could cause oral cavity and oropharyngeal cancer?” “How do you imagine oral cavity and oropharyngeal cancer looks like in the mouth?” “Can oral cavity and oropharyngeal cancer manifest without initial complaint, pain, or symptom?” and “Is oral cavity and oropharyngeal cancer a contagious disease?”*. There were also Likert scale-styled questions that asked whether students thought HPV infection, tobacco and alcohol use, eating spicy foods, and exposure to sunlight increased an individual’s chance of developing oral cavity and oropharyngeal cancer. Answers in the knowledge portion of the questionnaire were then scored as 1 for ‘correct’ and 0 for ‘incorrect’. Each student’s answers were summed to create a scale, “knowledge score.”

#### Risk assessment

There were 4 risk perception questions on a 5-point Likert scale, and participants were asked to compare themselves to other smokers or drinkers of the same age and sex to describe what they thought their chances of developing oral cavity and oropharyngeal cancer in the future were, from most likely to least likely. There was also a question that assessed perceived severity of a potential cancer lesion: *“What would you do if you had a painless, abnormal swelling in your mouth for more than 2 weeks?”*

#### Data analysis

Our final sample size represents a convenience sample of 100 students out of 115 students approached to take the survey, yielding a response rate of 86.96%. Outcome of interest was oral cavity and oropharyngeal cancer knowledge level, a continuous variable which was derived from the knowledge score formed. Pearson’s correlation, Chi-Square, and one-way ANOVA assessed bivariate associations between co-variables and outcome of interest. For the purpose of binary logistic regression analyses, knowledge score was categorized as low vs. high; low being knowledge score of 0 – 7, and high being 8 – 14. Crude measures of association was performed for all covariates in the data. Data was analyzed using SPSS version 20 (Chicago, IL, USA). A two-tailed alpha of .05 was applied as a standard of significance in all analyses.

## Results

**Fig. 1:**
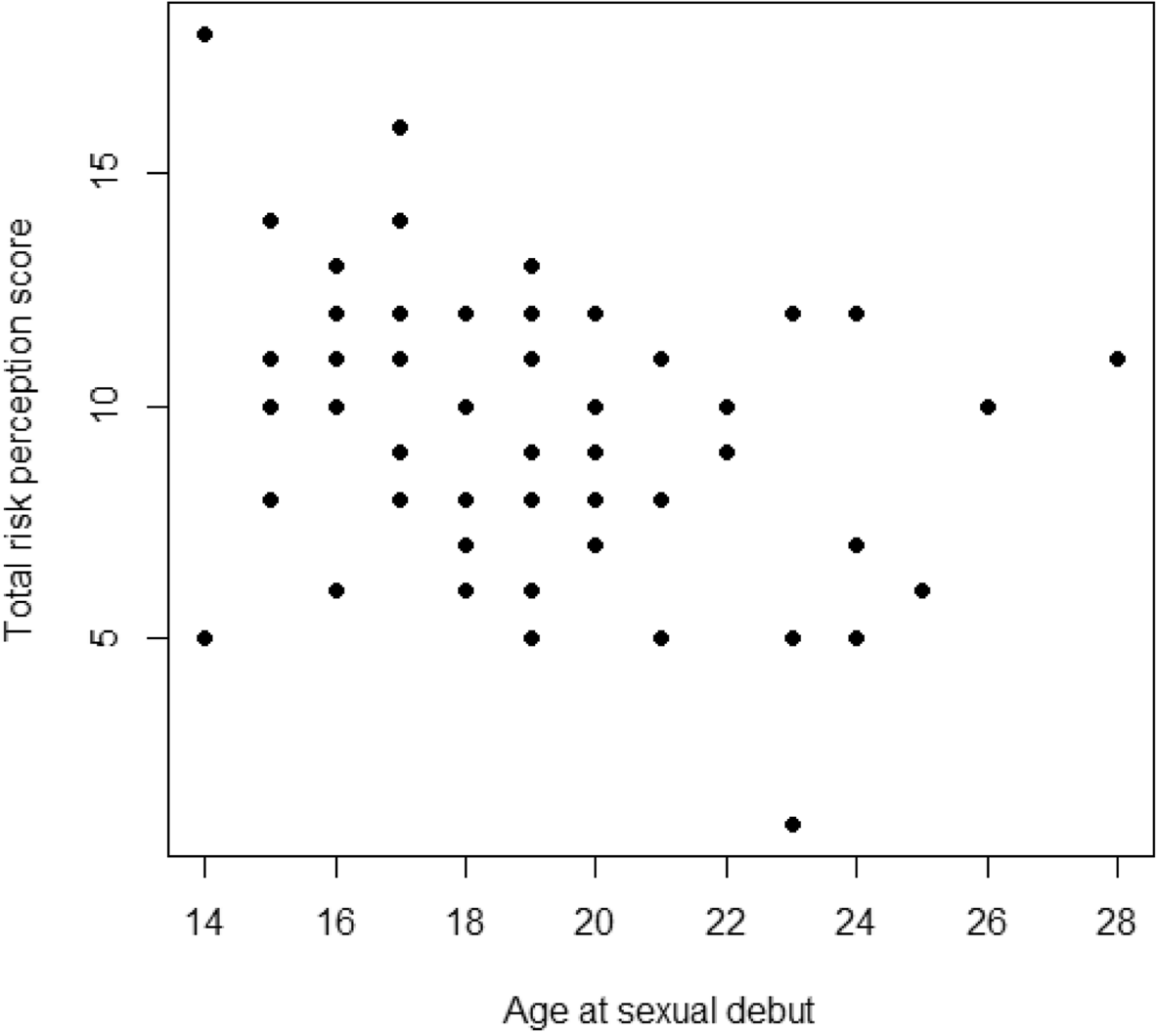
Correlation between students’' perception of risk and age at sexual debut. This figure shows a significant, negative correlation between age at sexual debut and risk perception of students

There were 100 participants in the study out of 115 students approached, yielding a response rate of 87%. There were 58% male students in the survey, and 46% of the students were undergraduates (See Table 1 for demographic characteristics of the population). Demographic data yielded 37% Caucasians, 37% Asians, and 19% African-Americans in the study. A majority of students (93 %) reported that they were non-smokers, while 35% were non-drinkers. Sixty-eight percent had initiated sexual intercourse, and the youngest age of sexual debut was 14 years old. For oral cavity and oropharyngeal cancer knowledge, 81% had a low score (between 0–7), while only 19% had a high score (8–14). Yet, only 2% perceived that their oral cavity and oropharyngeal cancer risk was high. Risk perception was negatively correlated with age at sexual debut (*r*(64) = −0.26, p = 0.037; see Fig. 1).

**Table 1:** Sociodemographic characteristics of study population (N = 100)

Characteristics	N	%
Race		
African American	19	19
Asian	37	37
Caucasian	37	37
Hispanic/Latino	3	3
Mixed	4	4
Age Groups		
16 - 20	23	23
21 -25	44	44
26 - 30	23	23
31& over	10	10
Gender		
Male	58	58
Female	41	41
Unknown	1	1
Smoking Status		
Smoker	3	3
Never smoked	93	93
Quit smoking	3	3
Trying to quit	1	1
Drinking status		
Drinker	65	65
Non-drinker	34	34
Stopped drinking	1	1
Marital Status		
Married	13	13
Single	87	87
Level of study		
Undergraduate	46	46
Masters	34	34
Doctoral	18	18
Other	2	2

There was no significant association between oral cavity and oropharyngeal cancer knowledge and perception of risk; however, knowledge was significantly associated with frequency of prevention of STDs

**Fig. 2:**
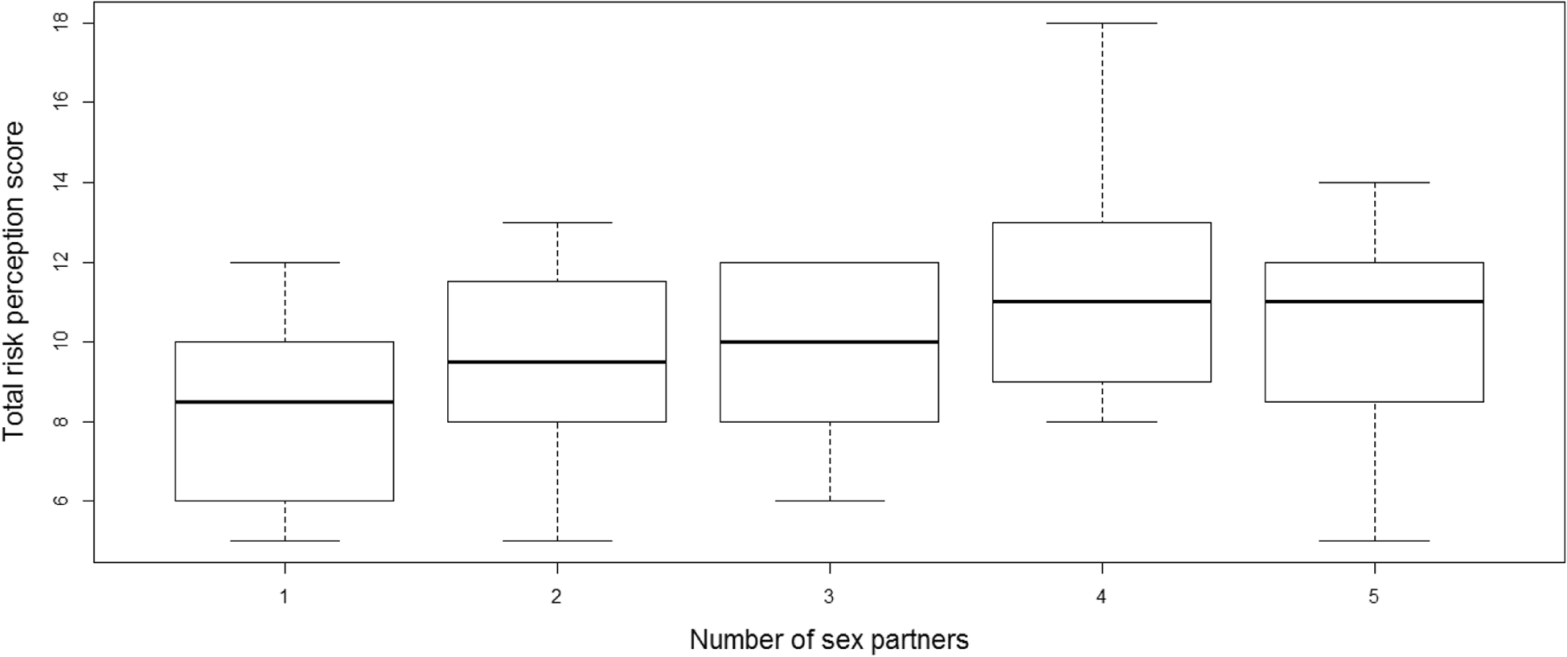
Association between number of sexual partners and perception of oral cavity and oropharyngeal cancer risk. This figure shows that students with 3 or more sexual partners were more likely to have a high risk perception

*F*(1, 65) = 4.90, p = 0.03, A one-way ANOVA showed a significant positive association between risk perception and number of sexual partners, *F*(4, 60) = 2.48, *p* = 0.05; see Fig. 2).

Although 86% claimed to have heard about oral cavity and oropharyngeal cancer, only 7% of these reported ever having an oral cavity and oropharyngeal cancer examination (See Table 2)**.**

**Table 2:** Oral cavity and oropharyngeal cancer and HPV knowledge questions

	Yes	No	Don't know
Characteristics	N (%)	N (%)	N (%)
Have you heard of oral cavity and oropharyngeal cancer?	86 (86)	12(12)	2 (2)
Have you ever heard of oral cavity and oropharyngeal cancer mouth exam?	18 (18)	66 (66)	16 (16)
Have you ever had an oral cavity and oropharyngeal cancer exam?	7 (7)	83 (83)	10 (10)
Certain types of HPV lead to oral cavity and oropharyngeal cancer	63 (63)	12 (12)	25 (25)
HPV is the same as HIV	1 (1)	76 (76)	23 (23)
Most types of HPV cannot clear up on their own	61 (61)	16 (16)	23 (23)
A person usually has symptoms when infected with HPV	14 (14)	62 (62)	24 (24)
Chance of getting HPV increase with number of sex partners	75 (75)	2 (2)	23 (23)
There is an HPV vaccine for both men and women	53 (53)	24 (24)	23 (23)

## Discussion

In the population we surveyed, only 3% of students who participated in this study reported they were current smokers. Previous studies have revealed that between 14% and 62% of university students may be considered smokers in the United States [23–27]. We note that this was self-reported data, and social desirability may lead to underreporting. However, another explanation could be that the university where the survey was conducted had implemented a comprehensive smoking ban on campus, and although we do not have information on smoking rates prior to the ban, it has been reported that such bans are associated with decreased smoking rates [28].

We found in our study that oral cavity and oropharyngeal cancer knowledge was not significantly associated with smoking or drinking rates, but was associated with a sexual risk factor for developing oral cavity and oropharyngeal cancer, and with frequency of prevention of STDs. It was also demonstrated that there were significant associations between risk perception and number of sexual partners, as well as the age of sexual debut. This is interesting finding, as literature shows that a significant proportion of young people who are experimenting with oral sex in fact consider oral sex less risky, and/or are more likely to have multiple oral sexual partners than vaginal sexual partners [29]. These sexual habits put them at a higher risk for developing oral cavity and oropharyngeal cancer, especially HPV-associated oropharyngeal cancer. It will remain crucial then to devise strategies to increase the awareness of cancer risks associated with sexual behavior. In this study, a majority of students self-reported that they have never heard of HPV. As HPV is a major driver of overall oral cavity and oropharyngeal cancer incidence, it will be important that information regarding HPV and sexual risk taking become an essential component of all oral cavity and oropharyngeal cancer prevention efforts. At least two-thirds of this study population may benefit from interventions stressing the need for HPV vaccines as part of the HPV vaccine catch up age range, which may hold some promise in not only preventing HPV strains that cause cervical cancer, but also oropharyngeal cancers [30, 31].

Only 7% reported to have had an oral cavity and oropharyngeal cancer examination. The American Cancer Society recommends that oral cavity and oropharyngeal cancer screening should be part of the periodic check for adults when they visit a dentist or other clinicians [32]. And while there remains insufficient evidence to accept or reject routine mouth screenings by primary care physicians in asymptomatic individuals, both the US Preventive Health Services Task Force (USPSTF) and the American Dental Association concede that dentists, otolaryngologists, primary care physicians and other clinicians may decide to screen high-risks groups, based on lifestyle factors or age, and those who may have reasons to suspect a lesion in their mouth [33–35]. This highlights the need for frontline healthcare providers, especially primary care physicians and dentists, to better understand the oral cavity and oropharyngeal cancer risk profiles of their patients, in order to reduce missed opportunities in the clinic for prevention of oral cavity and oropharyngeal cancer via regular oral cancer screenings and education.

There were some limitations in this study. Primarily, the study was non-experimental, and although Caucasians, African-Americans, and Asians were almost equally surveyed, there were few Latinos present in the study. Additionally, study participants stemmed from a single university, yielding a relatively small sample size. Thus, we may not be able to generalize results in the context of university students’ population in the United States. Data analysis may have revealed more robust associations between variables if the study had compared medical vs. non-medical students to test whether the assumption that medical students are likely to have better oral cavity and oropharyngeal cancer knowledge is true. Notwithstanding these limitations, this project has helped to generate baseline information on the amount of knowledge non-medical students possess regarding oral cavity and oropharyngeal cancer, as well as perception of their risk of developing oral cavity and oropharyngeal cancer. It may be the first study in the United States to exclusively assess oral cavity and oropharyngeal cancer knowledge and knowledge of risk factors (smoking, drinking, and HPV) among a university student population that does not include medical and dental students. Furthermore, in an age where HPV-associated head and neck cancers are increasing in epidemic proportions, our finding that sex-related oral cavity and oropharyngeal cancer risk factors may be more salient among university students than traditional risk factors, such as tobacco and alcohol, is hugely important for future educational interventions, and is worth exploring further.

## Conclusions

Oral cavity and oropharyngeal cancer knowledge and risk perception is low among this student population, and among the risk factors assessed in this population, it is to be concluded that sexual risks are more salient than the traditional oral cavity and oropharyngeal cancer risk factors of tobacco and alcohol use. Therefore, while tobacco cessation efforts and campus-wide smoking bans remain in place to continue addressing smoking rates, these efforts alone may not impact the prevalence of oral cavity and oropharyngeal cancer risk factors among university students. Increasing the awareness about other non-smoking related risk factors, especially those related to sexual behavior, may prove to be more effective in preventing oral cavity and oropharyngeal cancer among university-aged students. Inasmuch as health behavior is associated with risk perception, and oral cavity and oropharyngeal cancer incidence is increasingly shifting towards younger adults, interventions must be tailored to this group in order to improve prevention and control. Prevention of oral cavity and oropharyngeal cancers may pose a difficult challenge without first improving the knowledge of oral cavity and oropharyngeal cancers among high risk groups, particularly university-aged youth in the United States. In addition, since oral cavity and oropharyngeal cancer risk factors are largely prevalent among young adults, it may be of value to increase awareness of cancer risk factors and primary prevention strategies among elementary, middle and high school students, as many of the risk behaviors are likely to be initiated even before college age [36].
